# Mr. Hawkridge, on Diminished Excitement

**Published:** 1808-04

**Authors:** W. B. Hawkridge


### *To the Editors of the Medical and Physical Journal.*

GENTLEMEN,

ON reading your Journal for February, I observed a Paper written by Dr. Mease, on Cold Bathing, to rouse excitability. The practice of Dr. Mease, in the case he cites, evinces him, in my opinion, a man of superior practical knowledge. I have no motive whatever for offering any remark upon Dr. Mease's paper, but what may be supposed to arise from a constant and ardent wish to support the doctrine he appears to have espoused; the truths of which, unfortunately for mankind, are not by far so generally known and practised upon as the interesting subject of medicine demands: I mean, the Elementa Medicinæ Brunonis. Actuated by this desire, I feel extremely anxious that no misapplication of the terms, (so framed by the illustrious author, and used by its abettors,) should ever give its enemies an opportunity for displaying more spleen than they have hitherto done, by demonstrating, that even its friends do not sufficiently comprehend it, to enable them to practise, or write upon it with accuracy. Dr. Mease's Case, I think, is one of the happy instances that have occurred, being completely elucidative of the truth of Brown's principles: the remedies that were successfully used, evidently proving diminished excitement, consequently direct debility, or excess of excitability; therefore, instead of rousing excitability, Dr. Mease's plan of cure, was to exhaust or diminish, what by its redundance constituted the disease.

I am, &c.

W. B. HAWKRIDGE.

*March* 7, 1808.

